# A laboratory-based study on patients with Parkinson’s disease and seborrheic dermatitis: the presence and density of *Malassezia* yeasts, their different species and enzymes production

**DOI:** 10.1186/1471-5945-14-5

**Published:** 2014-03-14

**Authors:** Valentina S Arsic Arsenijevic, Danica Milobratovic, Aleksandra M Barac, Berislav Vekic, Jelena Marinkovic, Vladimir S Kostic

**Affiliations:** 1 Institute of Microbiology and Immunology, University of Belgrade Medical School, Dr Subotica 1, Belgrade, Serbia; 2 Department of Dermatology, Dermatology Unit, Clinical Center of Serbia; Military Medical Centre, Belgrade, Serbia; 3 Management School, Alfa University, Belgrade, Serbia; 4 Institutes for Statistics and Medical Informatics, University of Belgrade School of Medicine, Belgrade, Serbia; 5 Institute of Neurology Clinical Centre of Serbia, University of Belgrade, School of Medicine, Belgrade, Serbia

**Keywords:** Seborrheic dermatitis, Parkinson’s disease, *Malassezia* species, Colony forming units, Enzymes

## Abstract

**Background:**

Seborrheic dermatitis (SD) and Parkinson’s disease (PD) are frequently associated conditions. Aims of this study were: to determine severity of SD, presence of different species and density of *Malassezia* yeasts; to assess yeast lipases and phosphatases production *in vitro* and to compare these results between SD patients with and without PD.

**Methods:**

This case–control prospective study was conducted at the Dermatology and Neurology Units, Clinical Centre of Serbia and at the National Medical Mycology Reference Laboratory, University of Belgrade Medical School, Serbia. A total of 90 patients and 70 healthy controls (HC) were investigated: 60 patients with SD (SDN) and 30 patients with SD and PD (SDP). Culture-based mycological examination was carried out on lesional skin (LS) and non-lesional skin (NLS). A yeasts density was determined by counting the *Malassezia* colony forming units per tape (CFU/tape). Enzymes production by isolated *Malassezia* was investigated.

**Results:**

The most patients with SD were male (76.7%; SDP and 63.3%; SDN) and the intensity of SD was dominantly severe or moderate (76.7%; SDP and 75%; SDN). The presence of *Malasseziа* was high on LS in both groups (87.3%; SDP and 86.7%; SDN) (p=0.667).

The highest yeasts density (mean CFU/tape=67.8) was detected on LS in 53% of SDP group and in 21.7% of SDN group (mean CFU/tape=31.9) (p < 0.01). The presence of negative cultures was lower in SDP group (13.3%) in comparison to HC and SDN groups (37% and 31.7%, respectively). *Malassezia* density on NLS in SDP group (mean CFU/tape=44.3) was significantly higher in comparison to SDN and HC (p=0.018). *M. globosa* was the most abundant species identified amongst isolates from the SDP group (42.3%) and exhibited high production of phosphatase and lipase *in vitro*.

**Conclusion:**

From this laboratory-based study a positive correlation between SD, PD, *M. globosa* incidence, high yeast density and high phosphatase and lipase activity was established. Our data lead to conclusion that local skin performance of PD patient’s characterized with increased sebum excretion ratio play a role in SD by stimulation of yeasts replication and enzyme production.

## Background

Parkinson’s disease (PD) is the second most common neurodegenerative disorder which affects approximately seven million people globally and one million people in the United States [1]. The prevalence of PD is about 0.3% of the whole population in industrialized countries, 1% in the elderly over 60 years of age and 4% of the population over 80 years [2]. “Young-onset PD” appears between ages 21 and 40 years with greater probability for developing various adverse effects of treatment, and deterioration of the quality of life [3].

Patients with PD, in addition to motor system disorders, often suffer from non-motile symptoms, which can include decreased facial skin motility resulting in a characteristic hypomimic facial expression, which has an impact on their quality of life [4]. This condition is associated with seborrheic dermatitis (SD) and increased sebum excretion ratio (SER) in almost 60% of the cases most likely due to the parasympathetic system hyperactivity as well as to the systemic effect of Melanocyte-stimulating hormone (MSH) [4,5].

SD is chronic skin disease presented with typical sharply demarcated red patches and plaques with greasy scales in areas with increased density of sebaceous glands, namely the scalp, face, hairline, eyebrow, glabella, nasolabial folds, ears, upper trunk and flexures. SD affects approximately 3-5% of the population [6]. The intensity of symptoms, including inflammation and itching, varies with more severe clinical presentation occurring in predisposed groups. SD prevalence was found to be higher in some medical conditions including HIV positive and AIDS patients (34-83%) [7] and PD patients (52-59%) [8]. Endogenous and exogenous conditions, such as hormones (androgens), increased SER, altered immune response and neurological factors [9,10] were found to play an important role in the pathogenesis of SD, but the exact mechanisms are still to be elucidated.

Evident is recent increased research interest in lipophilic *Malassezia* yeasts. Genus *Malassezia* now contains 14 species, among which 7 are well characterized culturally and biochemically [11]. Members of this genus are part of normal human flora, however they are also thought to be exacerbating factor in a number of skin conditions, such as pityriasis versicolor, *Malassezia* folliculitis, SD, atopic dermatitis and even some life threatening nosocomial bloodstream infections [12,10]. *Malassezia* species and their skin density vary in different populations [13], countries, age groups and genders, as well as between normal, non-lesional skin (NLS) and lesional skin (LS) [14,11]. For currently unknown reason, these yeasts can change their saprophytic state and invade stratum corneum of the skin as pathogens.

Although SD is a multifactorial disease, there are two major factors in developing SD: the increased production of host sebum and the presence and increased reproduction of *Malassezia* yeasts. The presence and density of *Malassezia* yeasts, different species and production of enzymes in PD patients have not been investigated yet, thus the aim of this study was to determine the following: (I) the severity of SD in study group, (II) the presence and density of *Malassezia* and (III) enzyme production in isolated species in patients with SD and PD.

## Methods

### Source population

PD and SD patients treated at the Neurology and the Dermatology Units, Clinical Centre of Serbia from January to June 2011 were included in this study.

### Study population

There were 90 patients with SD, 21 to 82 years old, (61 men, 29 women; mean age 54 ± 17.5 years), among those 30 patients suffered from PD and SD at the same time (SDP group, 23 men, 7 women; mean age 67 ± 7 years) while 60 patients had SD only (SDN group, 38 men, 22 women; mean age 48 ± 18 years). Healthy control (HC) group were persons without SD (n = 60) randomly selected and matched by age and sex (44 men, 26 women; mean age 49 ± 19.5 years). HC had apparently normal facial skin, without any evidence of dermatitis. None of these patients had received topical or systemic antifungal therapy or corticosteroids for at least 4 weeks prior to sampling. All patients signed informed consents.

### Study design

This was a prospective case–control study which was conducted at the National Reference Medical Mycology Laboratory, Faculty of Medicine, University of Belgrade (laboratory-based investigation) and at Neurology and the Dermatology Units, Clinical Center of Serbia (clinically-based investigation). Determination of SD severity and laboratory-based investigation was done in order to determine the *Malassezia* yeast presence and their density, type and enzymes production.

### Determination of SD severity

A clinical assessment of SD was carried out and scored. The overall SD score was calculated by scoring the severity of SD (from 1 to 12) and the facial skin area affected (from 1 to 5). The severity of SD was evaluated according to the presence of: erythema, scale, infiltration and pustule. A four-point score was used for each parameter (0–absent, 1–mild, 2–moderate, 3–severe). The second scale was based on the percentage of the affected skin area: less than 10% (1 point), 10-30% (2 points), 30-50% (3 points), 50-70% (4 points) and over 70% (5 points). The result was obtained by multiplying the scores of both scales giving an overall score: mild SD (5 or less); moderate SD (6 to 11) and severe SD (from 12 to 60). Severity of SD has been described as: mild (the total score ≤5); moderate (the total score between 6 and 11); and severe (the total score between 12 and 60) [15].

### Sampling procedure, isolation and quantification of *Malassezia* yeast

Three skin samples were taken from SD patients’ foreheads, from LS and NLS, as well as from normal-looking skin from the foreheads of HC. The sampling was done by stripping 4 cm^2^ tape (Superabsorb® F, Lohmann Rauscher) [16]. Each tape was transferred to Dixon and Leeming*-*Notman agar (LNA) in order to isolate *Malassezia* lipophilic yeast and to Sabouraud agar in order to isolate non-lipophilic yeasts *M. pachydermatis* or *Candida*. Plates were incubated at 32°C for 10 days and observed daily. The cultures were considered positive for *Malassezia* if there was growth on Dixon and LNA. Yeasts isolates were quantified and identified using morphological and biochemical tests. The number of *Malassezia* colonies was counted and the growth densities were expressed as the mean number of colony forming units per tape (mean CFU/tape). The *Malassezia* density (CFU/tape) was performed of Dixon and LNA media and ranked into three levels: low (CFU/tape 1 ≤ 19), medium (CFU/tape =20-40) and high (CFU/tape > 40) [13]. Production of enzymes from the protease, lipase, phosphatase and glycosidase classes was determined for selected isolates.

### Identification of Malassezia yeasts

**Figure 1:**
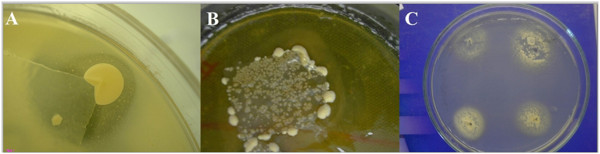
**Pictures of isolated *Malassezia *on culture.** Colony forming unit’s per tape (CFU/tape) expressed low density of *Malassezia* yeasts isolated on Leeming-Notman agar (LNA) from non-lesional skin (NLS) in healthy controls (HC) **(A)**; high density of *Malassezia* yeasts isolated on Dixon agar from lesional skin (LS) in Parkinson disease (PD) **(B)** and identification of *Malassezia* by Tween assimilation test **(C)**.

Seven lipophylic *Malassezia* species were identified*: M. globosa*, *M. furfur*, *M. slooffiae M. sympodialis, M. restricta, M. obtusa* and *M. japonicа* based on microscopic characteristics, presence of catalase and the ability of each isolate to utilize different Tweens [17]. The presence of catalase was determined by applying a drop of 10% hydrogen peroxide on a culture smeared on a glass slide. The production of gas bubbles indicated a positive reaction. The ability to utilize different Tween substrate was assessed on the agar plates, by spreading fresh yeast suspension onto plates. Tween 20, 40, 60 and 80 (5 μl) were added in 4 wells (2 mm diameter) in each plate. The plates were incubated at 32°C for one week and the growth of yeast around individual wells contain different Tween was recorded and *Malassezia* were identified (Figure 1).

### Determination of *Malassezia* yeasts enzyme production by ApiZym assay

The activity of 19 different enzymes produced by *Malassezia* was tested using the API Zym assay (bioMerieux, Marcy l’Etoile, France). A total of 26 *Malassezia* isolates from SD groups were tested for: proteases (n = 5), lipases (n = 3), phosphatases (n = 3), glycosidases (n = 8). Results were expressed semi-quantitatively, ranging from no activity to maximum enzymatic activity.

### Data collection

All subjects had a complete history, physical examinations and necessary procedures from skin were done to confirm the diagnosis. Trained mycologists carried out laboratory analysis and were engaged in data collection and data entry.

### Data analysis

Statistical analysis was done using the Statistical Package for Social Science (SPSS) program version 15.0. T-test was used to analyze the differences between severity of disease and demographic data; Mann–Whitney and Kruskal Wallis tests were used to analyze the median number of *Malassezia* CFU/tape within the same groups on LS and NLS and the median number of *Malassezia* CFU/tape from all patients with SD and HC. Chi-square tests were performed to determine the differences in: (I) the prevalence of different species of *Malassezia* in SDN and SDP groups, (II) the prevalence of different species of *Malassezia* in all patients with SD and HC. The relationship between study groups and culture-based mycological findings was determined by logistic regression analyses adjusted on sex and age.

The study was approved by the Ethical Board of the School of Medicine, University of Belgrade, and the Clinical Center of Serbia (No 5030/5). The anonymity of individuals was preserved during the study.

## Results

### Patient’s demographic data and determination of SD severity

Most SD patients were male (77%; SDP and 63%; SDN) (p < 0.01) and intensity of SD was dominantly in severe or moderate form (76.7%; SDP and 75%; SDN). Taking into account SD categories, there was no statistically significant difference between SDP and SDN groups in terms of SD severity: mild (23.3%; SDP and 25%; SDN); moderate (60%; SDP and 45%; SDN); severe (17%; SDP and 30%; SDN) (p = 0.175) (Table 1). However, when SD was measured by scale there was a significant difference between SDP and SDN patients (OR 0.81; 95%CI = 0.66-0.99; p = 0.043).

**Table 1:** Demographics and description of SD

**Variables**	**Healthy Control (HC) n = 70**	**SDN n = 60**	**SDP n = 30**	**Total n = 160**	**p**
**Demographics**					
Sex M/F (%)	26/44 (37.1)	38/22(63.3)	23/7 (76.7)	87/73 (54.4)	0.000
Age	48.7 ± 19.9	48.3 ± 17.9	66.6 ± 6.3^*^	51.9 ± 18.7	0.000
**Description of SD**					
Intensity	-	8.65 ± 6.7	6.9 ± 2.7	8.1 ± 5.7	0.175
Intensity in categories					0.314
Mild (≤5)	-	15 (25)	7 (23.3)	22 (24.2)	
Moderate (>5 and < 12)	-	27 (45)	18 (60)	45 (50)	
Sever (≥ 12)	-	18 (30)	5 (16.7)	23 (25.6)	

*Abbreviations*: *HC*, Healthy controls; *SD*, Seborrheic dermatitis; *SDN*, Seborrheic dermatitis in patients without Parkinson disease; *SDP*, Seborrheic dermatitis in patients with Parkinson disease; *M*, Male; *F*, Female; *p<0.05.

### The correlation between presence and growth density of *Malassezia* yeasts on LS and NLS

Comparison of *Malasseziа* counts (CFU/tape) on LS and NLS between studied groups was carried out. The correlation between negative cultures and growth density of *Malassezia* yeasts on LS and NLS is shown on Table 2. The presence of *Malasseziа* was high in LS in both groups (87.3%; SDP and 86.7%; SDN) (p = 0.667). The highest yeasts density (mean CFU/tape = 67.8) was detected on LS in 53% of SDP group and in 21.7% of SDN group (mean CFU/tape = 31.9) (p < 0.01). The mean CFU/tape on NLS was the highest in the SDP (mean CFU/tape = 44.3) followed by SDN (mean CFU/tape = 19.8) and HC (mean CFU/tape = 17.4) (p < 0.01). Correlation between studied groups in *Malassezia* CFU/tape on NLS highlighting significantly higher *Malassezia* density in SDP group (p = 0.018) (Table 2).

**Table 2:** **Comparison of *Malassezia *** **colonies number (CFU/tape) on lesional and non-lesional skin between studied groups**

**The characteristics of experiment**					
**LS**	**HC n = 70**	**SDN n = 60**	**SDP n = 30**	**Total n = 160**	**p**
Number of colonies (CFU/tape)	-	31.9 ± 38.1	67.8 ± 51.7	43.9 ± 46.1	0.006
Number of colonies in categories	-				
None	-	7 (11.7)	4 (13.3)	11 (12.2)	
Low CFU/tape (≤20)		30 (50)	3 (10)	33 (20.6)	
Medium CFU/tape (20–49)	-	10 (16.7)	7 (23.3)	17 (10,6)	
High CFU/tape (≥ 50)	-	13 (21.7)	16 (53.3)	29 (18.1)	
**NLS**					
Number of colonies (CFU/tape)	17.4 ± 30.1	19.8 ± 31.1	44.3 ± 44.7^**^	23.4 ± 34.9	0.018
Number of colonies in categories					
None	26 (37.1)	19 (31.7)	4^***^ (13.3)	49 (30.6)	
Low CFU/tape (≤20)	27 (38.6)	23 (38.3)	11 (36.7)	61 (38.1)	
Medium CFU/tape (20–49)	9 (12.9)	9 (15)	4 (13.3)	22 (13.7)	
High CFU/tape (≥ 50)	8 (11.4)	9 (15.0)	11 (36.7)	28 (17.5)	

*Abbreviations*: *HC*, Healthy controls; *SDN*, Seborrheic dermatitis in patients without Parkinson disease; *SDP*, seborrheic dermatitis in patients with Parkinson disease; *LS*, Lesional skin; *NLS*, Non-lesional skin; *CFU*, Colony forming units; ^**^p < 0.05; ^***^p <0.01.

### The prevalence of different *Malassezia* species and their enzyme production

**Figure 2:**
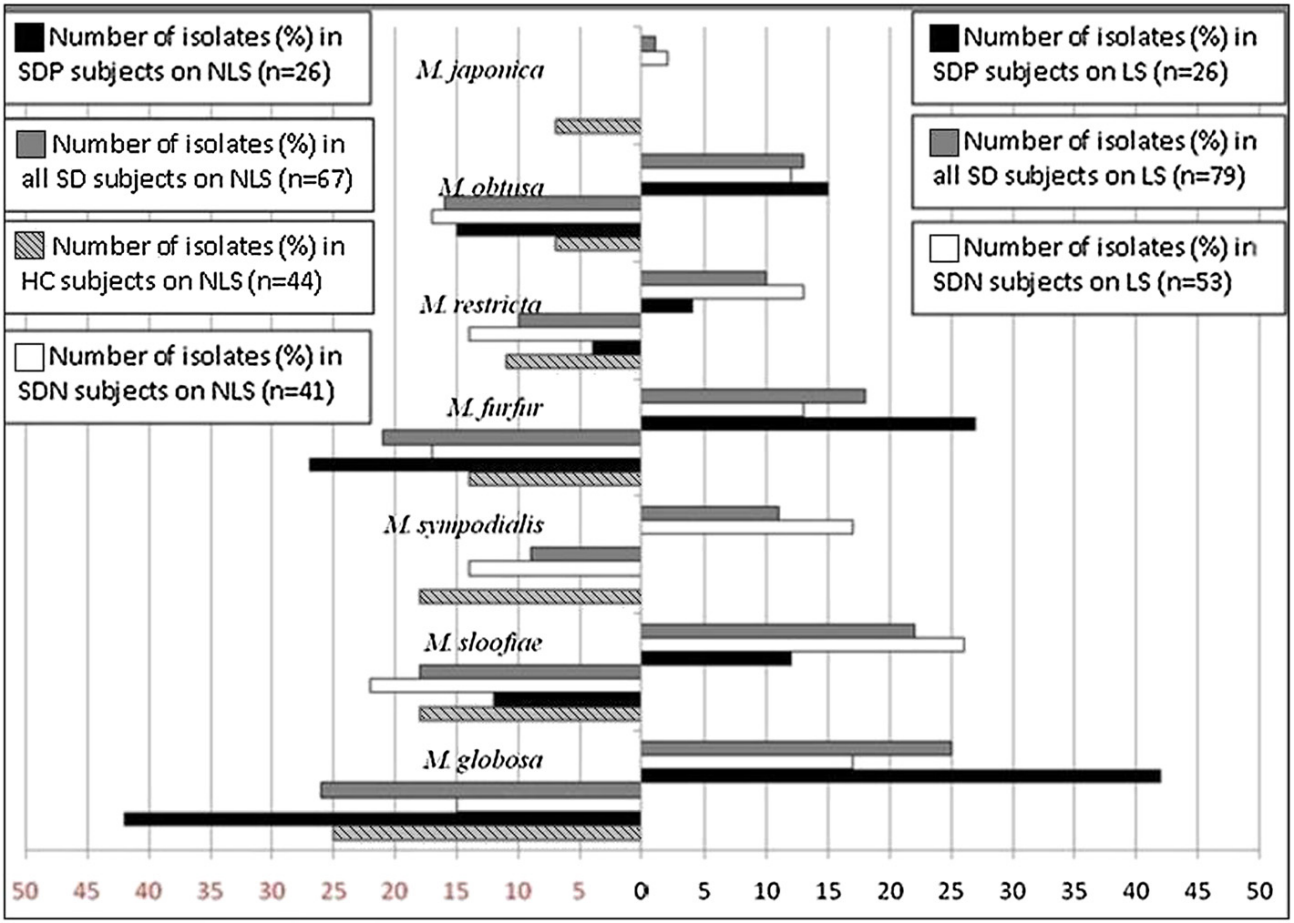
**Quantitative graphical presentation of different *Malassezia *species.** Graphical presentation based on the percent of total positive culture on LNA and Dixon media, isolated from non lesional skin (NLS) and lesional skin (LS) on the: (i) all patients with SD; (ii) patients with SD and without Parkinson’s disease (SDN); (iii) patients with SD and Parkinson’s disease (SDP) and (iv) healthy controls (HC).

The prevalence of different *Malassezia* isolated from NLS and LS in studied groups is shown in Figure 2. The most prevalent species in SDP/LS was *M. globosa* 11/26 (42.3%), followed by *M. furfur* 7/26 (26.9%) and *М. obtusа* 4/26 (15.4%). In SDN/LS the most prevalent species was *M. slooffiae* 14/53 (26. 5%), followed by *M. sympodialis* 9/53 (17%) and *M. globosa* 9/53 (17%) (p = 0.023). The differences were noted only for *M. globosa* (42.3%; SDP/LS) and *M. sloffiae* (26.4%; SDN/LS) (p < 0.05).

On NLS the most common species were: *M. slooffiae* (22%) in SDN group; *M. globosa* (25%) in HC and *M. globosa* (42.3%) in PD group. In SDP group the most prevalent species (*M. globosa, M. furfur* and *M. obtusa*) were the same on LS and NLS. The observed difference between isolated *Malassezia* on LS vs. NLS was not statistically significant for SDN and HC groups (SDN; p = 0.933), (HC; p = 0.541) (Figure 2).

The observed data suggest a significant role of infection, presence of different *Malassezia* species and yeast density in ethiopathogenesis of SD. Therefore, we evaluated the hypothesis that *Malassezia* yeasts replication and lipases production on the host skin may be important for pathogenesis of SD. Thus we tested (selection of isolates, n = 26) for production of different enzyme classes *in vitro*. All phosphatase and lipase enzymes were produced in significant amount from all tested *Malassezia* isolates. High rate of β–glucuronidase and leucine arylamidase activity was detected at *M. furfur* isolates only (Table 3).

**Table 3:** **Enzymatic activity of different *Malassezia *** **species determined by the Api Zym assay**

**Class of enzymes**	**Type of enzymes**	**All strains n = 26 (%)**	***M. furfur*** **n = 9 (%)**	***M. globosa*** **n = 7 (%)**	***M. slooffiae*** **n = 4 (%)**	***M. obtusa*** **n = 6 (%)**
Proteases	Leucine arylamidase	11 (42)	7 (78)	2 (29)	1 (25)	1 (17)
	Valine arylamidase	ø	ø	ø	ø	ø
	Cystine arylamidase	ø	ø	ø	ø	ø
	Trypsin	ø	ø	ø	ø	ø
	α - Chymotrypsin	ø	ø	ø	ø	ø
Lipases	Lipase (C14)	14 (54)	6 (67)	4 (57)	3 (75)	2 (33)
	Esterase lipase (C8)	25 (96)	9 (100)	7 (100)	4 (100)	5 (83)
	Esterase (C4)	25 (96)	6 (67)	4 (57)	3 (75)	2 (33)
Phosphatases	Acid phosphatase	26 (100)	7 (100)	7 (100)	4 (100)	6 (100)
	Alkaline phosphatase	18 (69)	7 (78)	4 (57)	3 (75)	3 (50)
	Naphthol-AS-BI- phosphohydrolase	23 (89)	8 (89)	7 (100)	4 (100)	5 (83)
Glycosidase	β – Glucuronidase	9 (35)	3 (33)	2 (29)	2 (50)	2 (33)
	α - Galactosidase	ø	ø	ø	ø	ø
	β - Galactosidase	ø	ø	ø	ø	ø
	N -acetil-β-glucosaminadase	ø	ø	ø	ø	ø
	α - Glucosidase	ø	ø	ø	ø	ø
	β - Glucosidase	ø	ø	ø	ø	ø
	α - Mannosidase	ø	ø	ø	ø	ø
	α - Fucosidase	ø	ø	ø	ø	ø

Data showed all type of enzymes from lipase and phosphate classes detected in a high percentage from the tested species isolated in this study.

### Correlation between study groups and culture-based mycological findings

The correlation between studied groups and culture-based mycological findings performed by logistic regression analysis for sex and age showed significant higher intensity of SD in SDN group (OR 0.81; 95% CI = 0.66-0.99; p = 0.043) (Table 4). Our data demonstrated predominant male sex in patients with SD and moderate or severe form of SD in both studied groups. There was no difference between studied groups in SD severity regarding categories, but when SD was measured by scale SD severity was a significantly higher in SDN group (logistic regression analyses for sex and age) (Table 4). Therefore, a positive correlation between SD, PD, *M. globosa*, high yeast density and high phosphatase and lipase activity of *Malassezia* yeasts was observed. This may suggest the possible role of the skin SER in PD patients for yeasts replication and lipases enzyme production.

**Table 4:** **Correlation between study groups and culture-based mycological findings expressed as number and presence of *Malassezia *** **colonies on lesional and non-lesional skin**

**Variables/potential classificatory of selected groups**	**Logistic regression analysis adjusted on age and sex (OR; 95% CI; p)**		
	**SDN vs. SDP**	**HC vs. SDN**	**HC vs. SDP**
Intensity of SD	0.81; 0.66-0.99; 0.043*	-	-
Intensity of SD in categories	0.46; 0.20-1.04; 0.063	-	-
Number of colonies (CFU/tape) on LS	1.01; 0.99-1.02; 0.110	-	-
Number of colonies (CFU/tape) on LS by categories	1.51; 0.62-3.64; 0.363	-	-
Number of colonies (CFU/tape) on NLS	1.01; 0.99-1.02; 0.398	0.99; 0.98-1.01; 0.786	1.01; 0.99-1.02; 0.288
Number of colonies (CFU/tape) on NLS by categories	1.35; 0.56-3.29; 0.504	0.98; 0.54-1.78; 0.954	1.60; 0.75-3.42; 0.225
Presence of *Malassezia* on LS	0.89; 0.17-4.68; 0.898	-	-
Presence of *Malassezia* on NLS	1.89; 0.45-8.02; 0.387	0.97; 0.44-2.12; 0.933	2.32; 0.63-8.53; 0.206

*Abbreviations*: *OR*, Odds ratio; *CI*, Confidence interval; *SD*, Seborrheic dermatitis; *SDN*, Seborrheic dermatitis in patients without Parkinson disease; *SDP*, Seborrheic dermatitis in patients with Parkinson disease; *HC*, Healthy controls; *LS*, Lesional skin; *NLS*, Non-lesional skin; *CFU*, Colony forming units; *p<0.05.

## Discussion

The concept of skin as a mirror of Parkinsonism dates back to the beginning of the last century. Since then, a good deal of evidence has been accumulated in support of the causal association between the neurological disturbance and changes detectable on areas of the integument with the richest sebaceous gland supply. SD is a common chronic and relapsing inflammatory skin disorder which primarily affects sebum rich areas. The prevalence of SD is 11.6% in USA [6] but can be higher in elderly people (over 80%) [5] and in Parkinsonism (52–59.5%) [8]. In recent years, a few studies concerning SD and PD have been published [18-20]. To our best knowledge, this is the first culture-based epidemiology study performed on patients with SD and PD. In the present study male SDP was dominant and this was in agreement with the study carried out by Gupta who explained the observed result by stronger action of androgens in the male SD patients [20], as well as with the study by Wooten who found a male to female ratio in PD of 1.49 [21]. Mean age of our SDP patients was 67 ± 7 years (Table 1) which was in agreement with studies restricted to individuals 65 years or above, confirming that the median incidence rate of PD was considerably higher in group above 60 year old [22].

The severity of SD depends on medical conditions and underlying diseases [13,11]. Accordingly, we observed a high number of SDP with moderate or severe SD form and a high number of positive cultures (Table 2). Our data could be explained by predominant male gender and an increased level of SER and MSH secretion in PD patients [23].

Our culture-based study demonstrated six different *Malassezia* isolated in SDP patients, with *M. globosa* (42.3%) as the most dominant species (Figure 1). Several studies identified *M. globosa* as the most prevalent species in SD, suggesting its principal role in SD [24] but data for patients with PD have not been reported yet. By comparing the severity of SD and *Malassezia* presence on LS we did not find a significant difference between studied groups. By contrast, using the laboratory based quantitative test for *Malassezia* yeasts density (expressed as CFU/tape) we found more than twice higher CFU/tape for SDP/LS than SDN/LS group. Literature data are controversial; some studies showed that the density increases with the intensity of skin lesions, while other studies were unable to confirm this result [10]. However, the reduction of *Malassezia* yeast density always results in SD outcome improvement [14].

The effects of the anti-Parkinson’s agent L-dopa on SD have been reportedly evaluated in Parkinson’s and data showed that treatment with L-dopa restores MHC-inhibiting factor synthesis and reduces sebum secretion in Parkinson’s [25]. Our patients were treated with L-dopa during the course of this study so this can be reason for the similarity in the clinical findings between different groups, even high SD intensity in SDN group (Table 4). However, other factors contribute to SD development such as facial immobility (“mask-like face”) which may lead to an increased sebum accumulation.

The pathogenesis of SD is still not completely understood but some clinical studies indicated that *Malassezia* presence and replication plays a pivotal role. Positive clinical response of SD to antifungals (i.e., azoles, ciclopirox) could be explained by their role in reduction of *Malassezia* yeast proliferation, again confirming an important role of *Malassezia* density and infection in pathogenesis of SD.

*Malassezia* requires exogenous specific lipids for growth, therefore tends to appear on skin around the time of puberty, when there is an increase in androgens and consequently an elevation in sebum production [12]. In patients with SD triglycerides and cholesterol are usually elevated but squalene and free fatty acids were significantly decreased compared to normal controls. Free fatty acids are formed from triglycerides through the action of bacterial lipases such as *Propionibacterium acnes*. This suggests that an imbalance of microbial flora and alterations in the composition of skin surface lipids may be involved [26].

*Malassezia* represents a part of human flora normally colonizing the skin surface but in some conditions it can change its saprophytic state and invade stratum corneum. Some culture-based laboratory studies identified differences between NLS and LS in the percentage of negative culture or in the presence and density of *Malassezia*[13]. We did not find significant difference in the absence of *Malassezia* on NLS between HC and SDN, but very low number of negative *Malassezia* cultures was detected on NLS in SDP. It may suggest an important role of the host environment such as the SER in PD group which may provide favorable conditions for expressing higher virulence capacity of *Malassezia*. Recent study showed a possible role of lipase in the host environment to produce free fatty acids, which are important for enhancing the *Malassezia* virulence [27]. The mechanisms of the *Malassezia* transition from commensal to pathogen are not clear yet, but this evidence is important for further studies due to the fact that lipases are further involved in the release of arachidonic acid, which can be important in cutaneous inflammation and disease [28].

It is well known that the development of infections depends on microbe’s replication while the severity of disease may depend of microbe’s enzyme production. In pathogenicity of SD phosphatase and lipase yeasts enzymes can initiate inflammatory response by releasing oleic and arahidonic acids from the sebum lipids [12,28]. Fatty acids have direct irritant and desquamative effects on keratinocytes, while arahidonic acid metabolized by cyclooxigenase serves as a source of pro-inflammatory eicosanoides and leads to inflammation and damage to stratum corneum. Our strains tested for different enzymes classes’ expressed all type of phosphatase and lipase enzymes manly at a high level (Table 4). These findings suggest that the infection, yeasts replication and density are key pathogenic mechanisms in SD and can contribute to the development of *Malassezia-*driven pathogenic “vicious circle”, which closes due to the fact that saturated fatty acids, released by *Malassezia* lipase, are used as a “proliferative fuel” for these yeasts. *M. globosa* showed the highest lipase activity, suggesting that lipase could be a pathogenic factor in the skin diseases associated with different *Malassezia* and providing an explanation about *M. globosa* as the most important pathogenic species in PD patients with SD [29].

Literature data suggest the role of another mechanism in pathogenicity of SD. Beside infection the allergy host reaction to *Malassezia* antigens can be involved in SD. In atopic patients, some enzymes from protease and glycosidase classes, such as β–glucuronidase and leucine arylamidase, may act as antigens. Thus, genetic predisposition, as well as *Malassezia* antigens can lead to the local skin immune response and immunopathology and to development of SD [12,29,30]. In our study we clearly demonstrated very low impact of enzymes which may act as *Malassezia* antigens (Table 3).

## Conclusions

This is the first culture-based epidemiology study performed on patients with SD and PD. The data demonstrated a positive correlation between SD, PD and *Malassezia* presence and density. Our findings that SDP group had high yeast density and associated high phosphatase and lipase activity *in vitro* may lead to conclusion that infection is the key mechanism of SD in PD patients. Future better understanding of the interactions between different *Malassezia* and PD host may provide new opportunities for improved SD control in PD. The appropriate antifungal treatment can be useful for PD patients. Antifungals may reduce *Malassezia* growth and their enzymes production overall improving patients well-being and quality of life. Correlation between reduction of *Malassezia* density, positive outcome of SD and the reduction of the non-motile symptoms in PD patients need to be further investigated.

## Abbreviations

SD: Seborrheic dermatitis; PD: Parkinson’s disease; SDP: Patients with SD and PD; SDN: Patients with SD without PD; HC: Healthy controls; LS: Lesional skin; NLS: Non-lesional skin; CFU/tape: Colony forming units per tape; SER: Sebum excretion ratio; MSH: Melanocyte-stimulating hormone.

## Competing interests

The authors declare that they have no competing interests.

## Authors’ contributions

VAA and DM were involved in study design, data collection, and analysis and results interpretation. DM and AB had done laboratory tests and data presentation. BV and JM were involved in data correlation and interpretation. VAA and VK were responsible for laboratory and clinical part of the study, had advisory and supervisory role and were responsible for important intellectual content. All authors drafted the manuscript and made substantial contributions to the revised manuscript. All authors read and approved the final manuscript.

## Pre-publication history

The pre-publication history for this paper can be accessed here:

http://www.biomedcentral.com/1471-5945/14/5/prepub
